# NKCC1 downregulation induces hyperpolarizing shift of GABA responsiveness at near term fetal stages in rat cultured dorsal root ganglion neurons

**DOI:** 10.1186/s12868-015-0180-4

**Published:** 2015-07-14

**Authors:** Joelle N Chabwine, Karel Talavera, Ludo Van Den Bosch, Geert Callewaert

**Affiliations:** Department of Cellular and Molecular Medicine, Katholieke Universiteit Leuven (KU Leuven), Louvain, Belgium; Neurology Unit, Department of Medicine, Faculty of Sciences, University of Fribourg, Chemin du Musée, 5, Fribourg, 1700 Switzerland; Laboratory of Ion Channel Research and TRP Channel Research Platform (TRPLe), Department of Cellular and Molecular Medicine, KU Leuven, Louvain, Belgium; Laboratory of Neurobiology, Experimental Neurology and Leuven Research Institute for Neuroscience and Disease (LIND), KU Leuven, Louvain, Belgium; VIB, Vesalius Research Center, KU Leuven, Louvain, Belgium

**Keywords:** Dorsal root ganglion neurons, GABA, Intracellular chloride, NKCC1, Bumetanide, Oxytocin, Fetal analgesia

## Abstract

**Background:**

GABA_A_ receptor-mediated neurotransmission is greatly influenced by cation-chloride cotransporter activity during developmental stages. In embryonic neurons Na–K–2Cl (NKCC1) cotransporters mediate active chloride uptake, thus increasing the intracellular chloride concentration associated with GABA-induced depolarization. At fetal stages near term, oxytocin-induced NKCC1 downregulation has been implicated in the developmental shift from depolarizing to hyperpolarizing GABA action. Mature dorsal root ganglion neurons (DRGN), however, express high NKCC1 levels and maintain high intracellular chloride levels with consequent GABA-induced depolarization.

**Results:**

Gramicidin-perforated patch-clamp recordings were used to assess the developmental change in chloride homeostasis in rat cultured small DRGN at the embryonic day 16 (E16) and 19 (E19). The results were compared to data previously obtained in fetal DRGN at E14 and in mature cells. A significant NKCC1 downregulation, leading to reduction in excitatory GABAergic transmission, was observed at E16 and E19.

**Conclusion:**

These results indicate that NKCC1 activity transiently decreases in DRGN at fetal stages near term. This developmental shift in GABAergic transmission may contribute to fetal analgesia and neuroprotection at birth.

## Background

Cation-chloride cotransporters largely determine the action of GABA during neurogenesis [1–5]. In immature neurons, Na–K–2Cl (NKCC1) mediates active Cl^−^ uptake, promoting depolarizing GABA action, whereas in adult neurons the Cl^−^-extruding KCC2 is generally considered to be involved in generating the hyperpolarizing effect of GABA. Mature dorsal root ganglion neurons (DRGN), however, express high NKCC1 levels and maintain high intracellular Cl^−^ concentration ([Cl^−^]_i_) with resulting GABA-induced depolarization [2, 6, 7]. This property is crucial for presynaptic inhibition of spinal sensory feedback [2, 7–11].

A NKCC1 downregulation has been observed around birth in different neuronal types of central nervous system (CNS) [2, 12, 13] as a result of circulating maternal oxytocin [14–16]. Previously, we showed that DRGN at embryonic day 14 (E14) displayed higher NKCC1 activity and higher intracellular [Cl^−^]_i_ levels than age-matched motor neurons (MN) [17]. Here, we investigated whether DRGN also display NKCC1 downregulation at fetal stages near term, before increasing again to high expression levels in adult stage [18, 19].

Our data show a marked decrease in DRGN [Cl^−^]_i_ at E16 and E19 compared to E14. Decreased activity of NKCC1 at E16 and E19 fully accounts for this reduction in [Cl^−^]_i_. A possible role for this transient shift in GABAergic responses in DRGN is discussed.

## Results and discussion

GABA-induced currents were measured in cultured E16 and E19 DRGN. The results were quantitatively compared to data previously obtained in DRGN at E14 [17].

**Figure 1:**
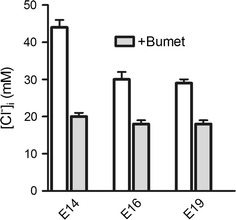
Intracellular [Cl^−^] and bumetanide-induced Cl^−^ reduction in DRGN. The resting [Cl^−^]_i_ (*open bars*) dropped significantly between E14 (44 ± 2 mM, n = 71) and E16 (30 ± 2 mM, n = 11, p = 0.00003), with no further decrease at E19 (29 ± 1 mM, n = 13, p = 0.81). In the presence of 10 μM bumetanide (+Bumet, *grey bars*), a specific blocker of NKCC1, [Cl^−^]_i_ was decreased to 20 ± 1 mM (n = 6) at E14 and to 18 ± 1 mM (n = 6) at E16 and E19. The bumetanide-insensitive component remained stable at all stages (p = 0.73 for E14 versus E16 and p = 0.46 for E14 versus E19).

Total membrane capacitance remained stable between E14 [17] and E16 at 24 ± 2 pF (n = 11, p = 0.43), and slightly increased to 30.7 ± 2 pF at E19 (n = 12, p = 0.006), indicating that most of the cells examined were small DRGN (capacitance 30 ≤ pF) [11]. Based on *E*_*GABA*_, [Cl^−^]_i_ markedly decreased from 44 mM at E14 to 30 and 29 mM at E16 and E19, respectively (Figure 1). In the presence of the selective NKCC1 blocker bumetanide (10 μM), [Cl^−^]_i_ was significantly reduced to ~20 mM at all stages, indicating that NKCC1-dependent Cl^−^ influx significantly dropped between E14 (57%) and E16 (33%), with no further decrease up to E19 (38%).

**Figure 2:**
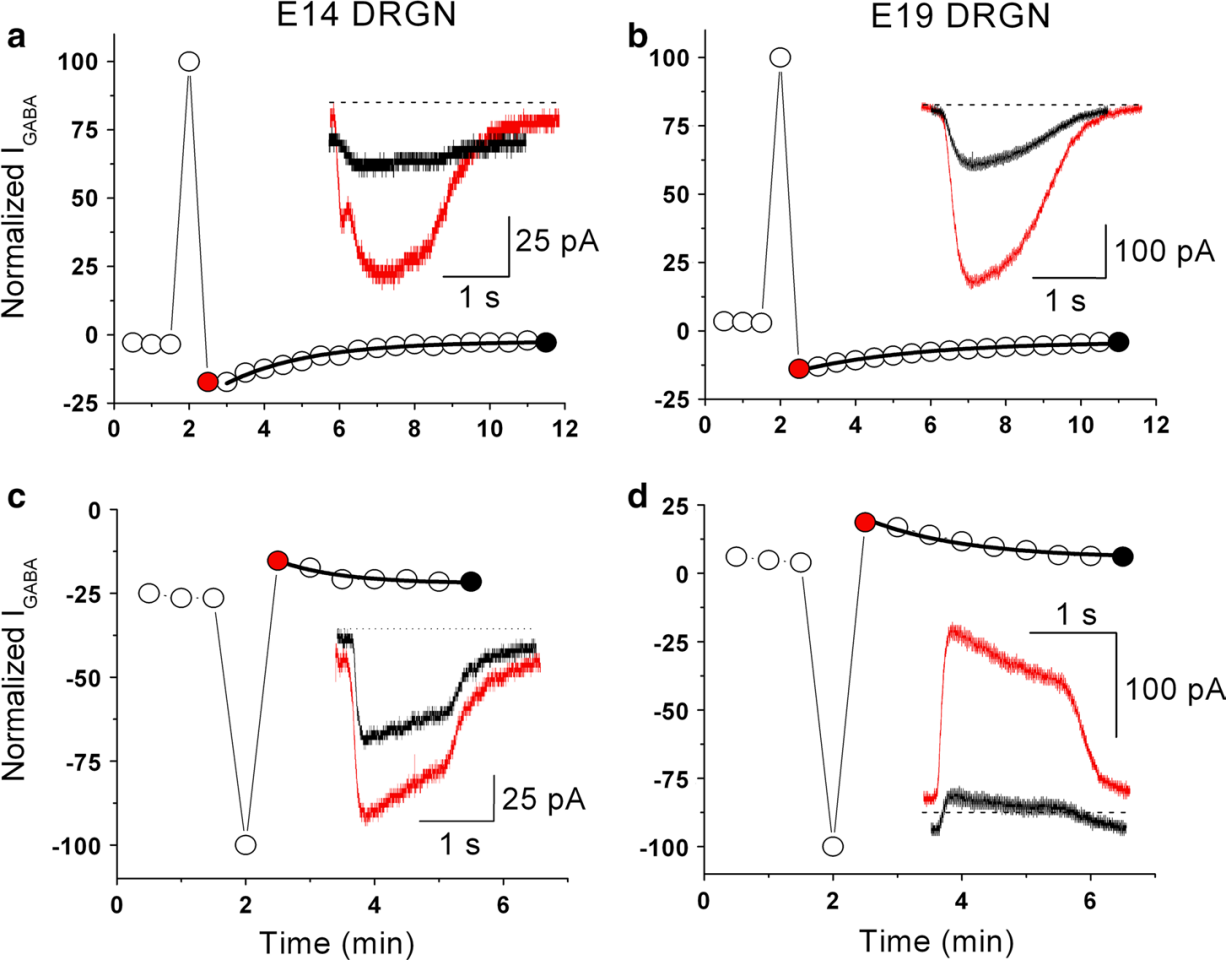
Cl^−^ load (**a**, **b**) and depletion (**c**, **d**) in DRGN at fetal stages E14 and E19. DRGN were held at −40 mV and 500 μM GABA was applied for 1 s every 30 s. Cl^−^ load was induced by a 5 s GABA pulse (1.5 mM) during a depolarizing step to +70 mV while Cl^−^ depletion was induced by applying the same pulse of GABA during a hyperpolarizing step to −100 mV. *I*_*GABA*_ was normalized to the value obtained during the voltage step to +70 or −100 mV. The *continuous lines* show the fit of data during recovery using a single exponential function. Recovery time constants after Cl^−^ load were 2.23 and 3.19 min at E14 (**a**) and E19 (**b**), respectively. Recovery time constants after Cl^−^ depletion amounted to 0.79 and 1.5 min at E14 (**c**) and E19 (**d**), respectively. *Insets* *I*_*GABA*_ recordings at times indicated by matched *colors.*

Changes in NKCC1 regulation were further explored by studying [Cl^−^]_i_ recovery after Cl^−^ load and Cl^−^ depletion in DRGN at E19. Applying 1.5 mM GABA for 5 s at a membrane potential of +70 mV consistently shifted the reversal potential in the positive direction, indicating an increase in [Cl^−^]_i_ from 33 to 43 mM (n = 2). Similar results were previously obtained in E14 DRGN [17] (p = 0.38 between respective increases in [Cl^−^]_i_). Following Cl^−^ loading, recovery to basal [Cl^−^]_i_ levels followed a single exponential function with a time constant of 2.97 ± 0.1 min (n = 5, Figure 2b), which is not different from that previously recorded in E14 DRGN [17] (Figure 2a, p = 0.44). Applying 1.5 mM GABA for 5 s at a membrane potential of −100 mV reduced [Cl^−^]_i_ by only 3 mM [from 31 to 28 mM (n = 3)] whereas in E14 DRGN, [Cl^−^]_i_ dropped by 8 mM. This difference in [Cl^−^]_i_ reduction between E14 and E19 DRGN during the depletion protocol was significant (p = 0.009). Following Cl^−^ depletion, recovery to the resting [Cl^−^]_i_ level was also significantly slower in E19 DRGN (time constant of 1.64 ± 0.1 min, n = 5) (Figure 2d) than in E14 DRGN (time constant of 0.9 ± 0.1 min) (p = 0.002, Figure 2c). Together, these data strongly suggest that NKCC1-related Cl^−^ fluxes markedly decreased in small DRGN after day E14.

It was previously shown that mature and embryonic DRGN sustain high [Cl^−^]_i_ owing to high NKCC1 expression [2, 11, 18]. Here, we show that embryonic DRGN undergo a significant downregulation of NKCC1 between E14 and E16 (maintained through E19), which significantly affects Cl^−^ homeostasis. The resulting overall decrease in [Cl^−^]_i_ limits the depolarizing action of GABA. Notably, [Cl^−^]_i_ at E14 is comparable to the level reported in the postnatal period (P0–P21) [19, 20] and possibly in adult DRGN [18] implying that the observed drop in [Cl^−^]_i_ between day E16 and E19 is only a transient phenomenon. The underlying mechanism most probably involves decreased NKCC1 activity rather than lower expression level [14, 20–22].

## Conclusion

These data accord with previous observations in other CNS neuronal types showing that NKCC1 activity is decreased as a result of increasing levels of circulating maternal oxytocin at near term. However, in contrast to other neuronal types, the activity-dependent downregulation of NKCC1 in DRGN is not permanent but only transient. Since we predominantly studied small DRGN (capacitances ≤30 pF) [11] in which GABA-induced depolarization is essential for sensory perception [2, 7–9, 11], we suggest that the transient GABAergic hyperpolarizing shift observed between E16 and E19 reflects oxytocin-induced fetal adaptation to delivery [14, 16], contributes to fetal analgesia and protects the fetus against neuronal insult.

## Methods

All experimental procedures were approved by the local Ethical Committee and were therefore performed in accordance with international ethical regulations. DRGN were derived from Wistar rat embryos at E16 and E19, and cultured as previously described [17]. Tissue samples were trypsinized and triturated and neurons were purified by centrifugation using a bovine serum albumin cushion and then plated on poly-l-ornithine and laminin-coated glass coverslips. Neurons were used between 3 and 6 days after plating. GABA-induced currents (*I*_*GABA*_) were recorded under voltage-clamp conditions using gramicidin-perforated patches [17] (75 μg/ml, Fluka). The pipette solution contained (in mM): CsCl 125, MgCl_2_ 1.2, HEPES 10, Na_2_ATP 2 and EGTA 1 and the pH was adjusted to 7.3 with CsOH. Patch pipettes (3–5 MΩ resistance) were briefly dipped in gramicidin-free pipette solution and back-filled with internal solution. After seal formation and stable access resistance ≤30 MΩ, GABA was locally applied using a fast perfusion system. The external solution contained (in mM): NaCl 150, KCl 6, MgCl_2_ 1, CaCl_2_ 3, HEPES 10 and glucose 10, pH adjusted to 7.3 with NaOH. 500 nM TTX (tetrodotoxin, Alomone labs) and 100 μM Cd^2+^ were added to block voltage-gated Na^+^ and Ca^2+^ channels, respectively. Total external [Cl^−^] amounted to 164 mM. Bumetanide (10 μM) was used to specifically block NKCC1 cotransporters [4, 6].

The reversal potential for *I*_*GABA*_, (*E*_*GABA*_) determined with brief voltage ramps during GABA application, was used to estimate intracellular Cl^−^ levels [17]. Intracellular [Cl^−^] depletion or loading was achieved by applying a 5 s pulse of GABA (1.5 mM) during a voltage step from the holding potential (−40 mV) to −100 mV or to +70 mV, respectively [12]. After depletion or loading, [Cl^−^] recovery was monitored by applying successive 1 s-GABA pulses of 500 μM every 30 s. [17]. All data are shown as mean ± SEM. Statistically significant differences were evaluated with unpaired Student t test (p < 0.05).

## Abbreviations

[Cl^−^]_i_: intracellular chloride concentration
CNS: central nervous system
DRGN: dorsal root ganglion neuron
*E*_*GABA*_: reversal potential for *I*_*GABA*_
EGTA: ethylene glycol tetraacetic acid
E14: embryonic day 14
E16: embryonic day 16
E19: embryonic day 19
GABA: gamma amino butyric acid
HEPES: 4-(2-Hydroxyethyl)piperazine-1-ethanesulfonic acid
*I*_*GABA*_: GABA-induced current
KCC2: potassium–chloride (K–Cl) cotransporter 2
MN: motor neuron
NKCC1: sodium–potassium–chloride (Na–K–2Cl) cotransporter 1
P0: post-natal day 0
P21: post-natal day 21
TTX: tetrodotoxin
